# Personality disorder: still the patients psychiatrists dislike?

**DOI:** 10.1192/pb.bp.115.052456

**Published:** 2017-02

**Authors:** Dimitrios Chartonas, Michalis Kyratsous, Sarah Dracass, Tennyson Lee, Kamaldeep Bhui

**Affiliations:** 1 Camden and Islington NHS Foundation Trust, London; 2 South London and Maudsley NHS Foundation Trust; 3 East London NHS Foundation Trust; 4 Queen Mary University of London

## Abstract

**Aims and method** In 1988, Lewis and Appleby demonstrated that psychiatrists hold negative attitudes towards patients with personality disorder. We assessed the attitudes of psychiatry trainees towards patients with borderline personality disorder and depression, expecting an improvement. 166 trainees were block randomised to receive one of four case vignettes that varied by diagnosis and ethnic group. We used Lewis and Appleby's original questionnaire and the Attitudes to Personality Disorder Questionnaire (APDQ).

**Results** We received 76 responses. Lewis and Appleby's questionnaire showed more negative attitudes towards personality disorder than depression, with no significant patient ethnic group effects, and the APDQ also showed a (weak) trend towards more negative attitudes to personality disorder. In subgroup analysis, only in the White British patient group were there significantly more negative attitudes to personality disorder. Factor analysis showed significantly less sense of purpose when working with personality disorder.

**Clinical implications** The perceived greater lack of purpose in working with personality disorder should be the target of clinical training and intervention. Targeted interventions that include training in managing personality disorder, supervision and practice in non-specialist, general psychiatry settings are important.

Personality disorder is characterised by an enduring dysfunctional and distressing pattern of inner experiences, behaviours and interpersonal interactions.^1^ Almost a quarter (24%) of people seen in primary care^2^ and 50% of people in secondary mental health services meet the criteria for a diagnosis of personality disorder.^3^ Patients with personality disorder have high rates of comorbidity^4^ and service utilisation.^5^ A meta-analysis, which identified 391 relevant publications and finally included 14, showed significant differences in prevalence between Black and White groups (although no differences between Asian or Hispanic groups compared with White groups), raising the question of whether there is a neglect of diagnosis in some ethnic groups.^6^

Although there is good evidence that borderline personality disorder (BPD) is a helpful diagnostic construct,^7^ some studies contest the validity (but not the clinical utility) of the diagnosis.^8^ Potter^1^ stresses that the psychological and interpersonal dynamics that patients with BPD bring to the clinical setting cause frustration and consternation among clinicians, such that BPD is now a diagnosis that can carry pejorative connotations and compound the stigma that such patients already face. Many patients with BPD feel rejected and disbelieved by clinicians.^9^ Improving clinicians' attitudes toward patients with BPD could bring them clinical benefit.^10^

Against this background, there have been systematic efforts to study clinicians' attitudes to personality disorder. A systematic review of the literature shows that healthcare professionals in general have negative attitudes towards such patients.^11,12^ A large study^7^ demonstrated that the clinician's occupational subgroup was significantly related to the attitude they adopted towards patients with BPD: staff nurses had the poorest self-ratings on overall caring attitudes, whereas social workers had the most caring attitudes. Social workers and psychiatrists self-rated highly on treatment optimism, whereas staff nurses rated poorly on empathy and treatment optimism. Overall, the authors suggested that education about the nature and treatment of BPD can lead to more positive attitudes, but also that clinicians with greater levels of experience in terms of both number of BPD patients and years of practice were more likely to express positive attitudes towards such patients. Bodner *et al*^13^ demonstrated that psychologists were less likely than psychiatrists and nurses to express antagonistic judgements towards patients with BPD and suicidal tendencies, whereas nurses were less likely to show empathy than psychiatrists and psychologists. They identified a number of factors influencing attitudes, including motivation to improve diagnostic skills, seniority, training and supervision, gender and familiarity with treatment modalities.

In 1997, Bowers *et al*^14^ developed a new instrument called the Attitude to Personality Disorder Questionnaire (APDQ). The APDQ is a reliable and valid measure of attitude to personality disorder, and is useful for outcome studies. It was tested among nurses and prison officers.^15–17^ One of these studies^16^ revealed that psychiatric nurses' attitudes were more negative than those of prison officers. Prison officers more often liked and showed an interest in prisoners with personality disorder, and they showed less fear and helplessness, less anger, more optimism about treatment and less frustration. The other studies found that nurses considered people with personality disorder difficult to treat; they were pessimistic about the efficacy and outcome of treatment and felt they were poorly trained to care for such patients.

Over 20 years ago Lewis & Appleby^18^ demonstrated that psychiatrists hold negative attitudes towards patients with a diagnosis of personality disorder, and that the diagnostic label was more influential on their attitudes than the patient's gender or class. Patients given a previous diagnosis of personality disorder were seen as more difficult and less deserving of care than controls. The authors concluded that a diagnosis of personality disorder carries enduring negative sentiments and is not used to guide effective treatment. For example, people with this diagnosis may experience difficulties when seeking help for psychiatric symptoms such as depression. The authors proposed that the concept of personality disorder be abandoned.

In 2002 the National Personality Disorder Development Programme was introduced in the UK, accompanied by the publication of *No Longer a Diagnosis of Exclusion*^19^ and *Breaking the Cycle of Rejection*,^20^ policy attempts that aimed to improve the evidence base of effective interventions for patients with personality disorder in specialist personality disorder services and, it was hoped, would also mitigate stigma.

Given the above, it seemed timely to see whether the attitudes of psychiatrists towards BPD showed any change. We selected psychiatric trainees, despite the difference in seniority/experience compared with Lewis & Appleby's original study (mean 16.5 years of psychiatric practice), as they are on the front line of service provision and are the next generation of consultant psychiatrists. Considering the findings of McGilloway *et al*^6^ and the multi-ethnic patient population in east and north-east London our sample was drawn from, we also examined the impact of ethnicity on attitudes.

## Objectives

The objectives of the study were:

- to assess the attitudes of psychiatry trainees towards patients with BPD compared with depression
- to assess the impact of patient ethnicity on the attitudes of trainees to BPD.

## Method

The study population consisted of the cohort of doctors training in psychiatry on the north-east London rotations (East London NHS Foundation Trust and North East London NHS Foundation Trust) between February and July 2013: core trainees 1–3, specialist trainees 4–6, general practice vocational trainees and foundation year 2 doctors. Overall, 166 doctors in training were block randomised in blocks of 8 to receive one of four different case vignettes (Box 1) that varied by previous diagnosis (BPD or depression) and ethnic group (White British or Bangladeshi). The case vignettes were modified versions of those used by Lewis & Appleby.^18^

**Box 1** Case vignettes used in the study

**Case 1**

A 25-year-old White British woman is seen in out-patients. She complains of feeling depressed and crying all the time. She is worried she may be having a ‘breakdown’ and is requesting admission. She says she has thought of killing herself by taking an overdose of tablets at home. She has a history of an overdose 2 years ago after a relationship break-up, following which she saw a psychiatrist who diagnosed her with depressive episode. She recently lost her job and is worried about how she will pay the bills. She is finding it difficult to sleep and her GP prescribed nitrazepam, which she says has been helpful and which she would like to continue.

**Case 2**

Same as case 1, but the patient is Bangladeshi.

**Case 3**

Same as case 1, but the previous diagnosis is borderline personality disorder.

**Case 4**

Same as case 3, but the patient is Bangladeshi.

We used the following tools to measure attitudes:

Lewis & Appleby's 22 semantic differentials on a 6-point scale. Using the original scoring conventions, the semantic differentials were scored so that a higher score represented responses that were more rejecting or that indicated lack of active treatment.

APDQ: a questionnaire that consists of 37 affective statements about patients with personality disorder (e.g. ‘I like personality disorder people’, ‘I feel drained by personality disorder people’, ‘I feel patient when caring for personality disorder people’). Respondents rate the frequency of their experiences of these feelings on a 6-point Likert scale: never, seldom, occasionally, often, very often and always. The responses can be summed to give a total score; the higher the score, the more positive the attitude towards patients with a personality disorder. Five subfactors can be scored:

- factor 1, enjoyment: warmth, liking for and interest in contact with patients with personality disorder
- factor 2, security: the lack of fears, anxieties and helplessness in relation to patients with personality disorder
- factor 3, acceptance: the absence of anger towards patients with personality disorder and a sense of being different from them
- factor 4, purpose: feelings of meaning and purpose in working with patients with personality disorder
- factor 5, enthusiasm: energy and absence of tiredness.

For the purposes of this study we modified the affective statements to ‘I like these patients’, ‘I feel drained by these patients’ etc. to correspond to the case vignette of either personality disorder or depression.

### Analysis

As regards Lewis & Appleby's 22-item semantic differentials, we compared mean and s.d. scores on items. We assessed the structure of the items by running a principal components analysis. We summed scores of the most dominant factors that explained most of the variance and compared them by diagnosis and by ethnic group.

The APDQ scores (mean, s.d.) were compared for trainees across the four case vignettes. These were compared as groups that differed by diagnosis or by ethnic group in logistic regression analyses, to assess the role of diagnosis and ethnic group. We used the original APDQ factors as an additional variable to assess differences by patient ethnic group and diagnosis.

The study was granted ethical approval by the South West London REC 3 (ref. 10/H0803/159). We obtained the names and positions of all trainees in the rotation from the core training scheme manager for the north-east London rotations. We contacted all trainees via email asking them to complete questionnaires online (on the SurveyMonkey platform, www.surveymonkey.co.uk). All respondents gave informed consent and all responses were anonymous.

## Results

We received 76 responses (response rate 46%). However, a small number of respondents failed to answer a number of questions. We thus analysed data from 73 responses to Lewis & Appleby's questionnaire (*n* = 19 for case 1, case 3 and case 4, and *n* = 16 for case 2) and 68 responses to the APDQ (17 for case 1, 15 for case 2, 20 for case 3, and 16 for case 4). Respondent characteristics are given in Table 1.

**Table 1:** Respondent characteristics

	Depression			Borderline personality disorder		
	British *n* = 20	Bangladeshi *n* = 16	Total *n* = 36	British *n* = 20	Bangladeshi *n* = 20	Total *n* = 40
Gender						
Female	12	10	22	13	8	21
Male	5	5	10	7	9	16
Unknown	3	1	4	0	3	3

Ethnicity						
White	8	6	14	11	9	20
Black/Asian/mixed/other	6	7	13	6	4	10
Unknown	6	3	9	3	7	10

Qualification in UK	10	8	18	11	8	19
Unknown	3	1	4	1	3	4

Current level						
GP/FY	2	5	7	4	0	4
CT1–3	10	5	15	11	12	23
ST4–6	5	3	8	5	5	10
Unknown	3	3	6	0	3	3

GP, general practice vocational trainee; FY, foundation year; CT, core trainee; ST, specialist trainee.

### Lewis & Appleby's 22-item semantic differentials

The scale was subject to principal components factor analysis followed by an orthogonal rotation to identify 16 of the 22 items loaded (loading of greater than 0.5) on the first factor (eigenvalue 10.42, explaining 71% of the variance), with two further candidate factors (eigenvalue 1.68, explaining 11.5% and eigenvalue 1.00, explaining 6.1%, respectively) (Table 2). Only items from the first factor were summed to compare attitudes, as the second and third factors were accounted for by 3 items each and did not show a clear conceptual distinction between each other. The mean and s.d. score of factor 1 was compared by diagnosis and by ethnic group (case 1: mean 42.42, s.d. = 8.54; case 2: mean 48, s.d. = 8.71; case 3: mean 53.68, s.d. = 11.99; case 4: mean 51.53, s.d. = 10.51). The scores did not vary by ethnic groups. The rank sums showed significant differences by diagnosis, with higher scores (more stigma) towards personality disorder than depression (overall Kruskal–Wallis χ^2^ = 11.38, d.f. = 3, *P* = 0.01) (Table 3).

**Table 2:** Principal components analysis

	Mean^a^ (s.d.)		Loading				
	Depression *n* = 35	BPD *n* = 38	Factor 1	Factor 2	Factor 3	Factor 4	Uniqueness
Factor 1 (eigenvalue 10.42)							
Poses difficult management problem	3.25 (1.18)	4.20 (1.30)	0.5955	0.0826	0.0343	0.1059	0.6261
Unlikely to improve	2.17 (0.94)	3.64 (1.48)	0.6828	0.2932	−0.0333	−0.4351	0.2574
Cause of debts under patient's control	3.67 (1.22)	3.28 (1.31)	0.6678	0.2539	0.1938	0.2264	0.4007
No mental illness	2.53 (1.38)	3.00 (1.57)	0.7153	0.2039	−0.041	−0.3236	0.3403
Case does not merit NHS time	2.64 (1.15)	2.97 (1.06)	0.6820	−0.2883	0.0797	0.1921	0.4085
Unlikely to complete treatment	2.67 (1.15)	3.95 (1.23)	0.7376	0.1704	−0.1875	−0.3877	0.2414
Unlikely to comply with advice and treatment	2.89 (0.95)	3.56 (1.27)	0.8410	0.1506	−0.0388	−0.2516	0.2052
Suicidal urges under patient's control	2.91 (1.00)	2.64 (1.40)	0.8697	0.0496	−0.1665	−0.0827	0.2066
Likely to become dependent on one	4.08 (1.11)	4.72 (0.79)	0.7435	−0.3069	0.2593	0.0553	0.2827
Condition not severe	3.25 (0.94)	3.54 (0.91)	0.8259	−0.3129	−0.1401	0.1625	0.1740
Admission not indicated	3.25 (1.50)	3.55 (1.40)	0.9096	0.0398	−0.1405	−0.0416	0.1496
Not a suicide risk	2.56 (0.99)	3.00 (0.99)	0.8246	−0.0480	−0.1447	0.2903	0.2126
Does not require sickness certificate	2.42 (1.59)	3.08 (1.51)	0.8481	−0.2232	0.0573	0.1831	0.1942
Dependent on BZs	3.29 (1.18)	3.08 (1.23)	0.8432	−0.2268	−0.0802	0.0578	0.2279
Psychotherapy referral not indicated	1.91 (1.16)	1.95 (1.11)	0.9452	−0.0484	−0.0436	0.0273	0.1017
Antidepressants not indicated	1.83 (1.16)	3.47 (1.59)	0.8676	−0.2914	−0.0017	0.1573	0.1377

Factor 2 (eigenvalue 1.68)							
Manipulating admission	2.91 (0.95)	2.68 (1.32)	−0.0609	0.6055	0.208	0.2771	0.5095
Unlikely to arouse sympathy	2.46 (1.09)	3.08 (1.36)	0.1055	0.6853	−0.1458	0.3179	0.3969
Would not like to have in one's clinic	2.86 (1.40)	3.36 (1.55)	0.3862	0.4406	0.1868	0.0396	0.6203

Factor 3 (eigenvalue 1.00)							
Taking an overdose would be attention seeking	2.97 (1.03)	3.64 (1.35)	0.2184	0.4940	−0.6602	0.1606	0.2466
Should be discharged from out-patient follow-up	1.61 (1.10)	1.82 (0.93)	0.3843	0.3520	0.6137	0.0264	0.3511
Likely to annoy	3.11 (1.28)	3.64 (1.48)	0.4816	0.1173	0.5391	−0.1399	0.4441

BPD, borderline personality disorder; BZ, benzodiazepine; NHS, National Health Service.

a. Means: higher values indicate greater agreement with statement; there was a 6-point scale between the two statements of the semantic differential.

**Table 3:** Attitudes to BPD based on the four test vignettes (factor 1: Kruskal–Wallis equality-of-populations rank test)

Case vignette	Respondents, *n*	Rank sum
1	19	460.50

2	16	564.50

3	19	860.50

4	19	815.50

χ^2^ = 11.38, d.f. = 3, *P* = 0.01

### APDQ

Multiple regression analysis of overall scores showed a weak trend towards lower scores in assessment of attitudes towards patients with a previous diagnosis of BPD compared with patients with a previous diagnosis of depression (lower scores indicate more negative attitudes in the APDQ and this is consistent with findings from the Appleby measure); however, this difference fell just short of statistical significance (*z* = 1.75, *P* = 0.08). There was no significant ethnic difference in attitudes towards patients. In subgroup analysis, only among White British patients with a previous diagnosis of BPD was there a lower overall score compared with White British patients with a previous diagnosis of depression (*z* = 1.98, *P* = 0.047).

This outcome had already been subjected to factor analysis by the original inventors of the measure. When we assessed scores on the basis of the five factors (using Kruskal–Wallis equality-of-populations rank) there was no statistically significant difference in scores for factors 1 (enjoyment), 2 (security), 3 (acceptance) and 5 (enthusiasm). However, there was a statistically significant (*P* = 0.03) difference found for factor 4 (purpose), with higher scores in attitudes (more positive) towards patients with depression (mean 4.60) compared with patients with a previous diagnosis of BPD (mean 4.15).

## Discussion

Since the original study of Lewis and Appleby nearly 30 years ago, a number of studies spanning from 1993 to 2012, as summarised in the introduction, have consistently shown that clinicians hold negative attitudes towards personality disorder. Our finding of more negative attitudes towards personality disorder compared with depression among psychiatric trainees, using the same instrument as Lewis and Appleby, is in line with previous research. However, it is difficult to show and theorise a sense of longitudinal change. This is mainly because different studies have looked at different professional groups, including nurses, prison officers, social workers, psychologists and psychiatrists, with varying training and levels of experience, and in different countries and/or care settings. In addition, our study examined the attitudes of a less experienced sample of psychiatrists than the Lewis and Appleby study, and this has to be taken into consideration when comparing current attitudes with previous ones. However, the ongoing finding of more stigma towards patients with personality disorder, almost 14 years after the introduction of the National Personality Disorder Development Programme, is disheartening.

More encouraging is the lack of evidence of differences in attitudes to patients with personality disorder of different ethnicity. The greater negative attitudes to personality disorder than depression in White British but not in Bangladeshi patients raises questions of differences in how clinicians may view the disorder in different ethnic groups, especially given that culture influences significantly what is considered to be a person and personality. Culture influences a number of factors relevant to the construct of personality disorder, such as learning inside and outside the family, the threshold when personality vulnerability cannot be compensated for by the person, and the social threshold when such decompensations are labelled pathological.^21–23^ If one accepts personality pathology as universal,^24^ perhaps this finding can also raise further questions regarding under-diagnosis of personality problems in certain ethnic groups, although supporting such a link is beyond the scope of this paper and further research is needed looking into both the universality of personality disorder and issues of under-diagnosis or misdiagnosis.

The question of why psychiatrists stigmatise personality disorder is complex and not simple to answer. In addition to the issues discussed above in relation to caring for these often emotionally draining patients, it is of relevance that specific features of BPD can cause negative attitudes. It is known that a wide range of impulsive and potentially self-damaging behaviours are observed, especially early in the course of the disorder.^25,26^ These include gambling, irresponsible money handling, reckless driving and unsafe sexual practices,^27^ as well as problematic substance use, self-harm, suicidal behaviour and disordered eating.^28–31^ Most of these behaviours carry strong moral connotations, sometimes challenging social norms, and can thus provoke negative reactions, triggering clinicians' implicit beliefs and possibly prejudices towards such behaviours.

While mounting anti-stigma campaigns may be required, the finding of a greater lack of purpose in clinicians in working with personality disorder allows for more modest and targeted intervention. Lack of purpose and therapeutic pessimism raise the importance of designing targeted interventions which may include training in personality disorder. As personality disorder is prevalent in all psychiatric settings, this is an important part of training for all psychiatrists.

### Limitations

Limitations of the present study include the small sample numbers, which, despite a reasonable response rate for a questionnaire study, makes it difficult to rely on comparisons between the groups, and thus compromises the power of the study. Our study population is taken from only two mental health trusts in the UK. However, the trusts cover both inner and outer London areas, and the training programmes are similar to those of others in the UK, as there is a specific framework for postgraduate training in psychiatry.

### Recommendations

Increased training in evidence-based practice for generalist mental health professionals in borderline personality disorder may address the issue of clinicians' lack of sense of purpose. The emphasis is thus on increasing the skills of clinicians in managing personality disorder in general psychiatric settings, which usually lack the structure, training and resources to deal with these complex patients. The difficulties faced by general psychiatry clinicians have been acknowledged in the literature, and in that respect ‘structured clinical management’ has been discussed as an effective way of working with BPD patients in non-specialist settings, as long as certain principles are followed and interventions implemented.^32^

It has been shown that people with personality disorder present specific challenges to the therapeutic alliance.^33–35^ Training and supervision^36–39^ as well as participation in a Balint group^40^ can improve negative attitudes.

Patients with personality disorder can provoke strong countertransference reactions, there is thus an ongoing need for clinicians to monitor their countertransference when working with such patients. This highlights the ongoing need for psychotherapy training. Evidence-based psychotherapy treatments have a documented applicability as a useful model for general psychiatrists.^41^ Supervision and further training is also necessary for consultants, as they often supervise trainee doctors and inevitably influence them through their own attitudes to these patients.

Recent research on stigma reduction has identified certain key ingredients that anti-stigma initiatives should take into consideration: a recovery emphasis and having multiple forms of social contact are especially important for maximising outcomes.^42^ These key ingredients can be taken up to introduce specific initiatives to reduce stigma against personality disorder. For example, Knaak *et al*^43^ found that a 3-hour workshop on BPD and dialectical behavioural therapy (DBT) was successful at improving attitudes and behavioural intentions towards persons with BPD. This is in line with those studies that show that training and education programmes tend to improve attitudes.
